# Xanthogranulomatous Osteomyelitis: A Systematic Review of Reported Cases, Diagnostic Challenges, and Treatment Outcomes

**DOI:** 10.7759/cureus.105725

**Published:** 2026-03-23

**Authors:** Adam W Youssef, Cameron Ballard, Dryden Dalbey, Paul M Doney, Rayhan Shaikh, Venkata S Puttagunta, Adil Bhatti, Michael D Hubbard

**Affiliations:** 1 Medicine, Kansas City University of Medicine and Biosciences, Joplin, USA; 2 Orthopedic Surgery, Mercy Clinic Orthopedics - Pittsburg, Pittsburg, USA

**Keywords:** bone infection, chronic osteomyelitis, foamy macrophages, tumor-mimicking lesions, xanthogranulomatous osteomyelitis

## Abstract

Xanthogranulomatous osteomyelitis (XO) is a rare form of chronic osteomyelitis characterized by mass-forming inflammatory lesions rich in lipid-laden macrophages. It commonly presents as an osteolytic, tumor-like bone lesion that may expand rapidly and mimic malignancy on imaging, leading to diagnostic uncertainty. This systematic review was conducted in accordance with Preferred Reporting Items for Systematic Reviews and Meta-Analyses (PRISMA) guidelines to characterize the clinical presentation, diagnostic strategies, treatment approaches, and outcomes of reported XO cases. Case reports and case series with histopathologic confirmation of XO were included. Extracted data encompassed patient demographics, immune status, anatomic involvement, clinical features, diagnostic modalities, management strategies, and outcomes. A total of 21 patients across 19 studies published between 1984 and 2025 were identified. The tibia (24%), rib (16%), and femur (12%) were the most commonly involved bones. Pain (81%) and swelling or mass formation (52.4%) were the predominant presenting features, while systemic symptoms were less frequent. Histopathologic evaluation established the diagnosis in all cases and was supported by radiographic and magnetic resonance imaging findings. Surgical intervention was performed in 76.2% of cases, whereas antimicrobial therapy was inconsistently administered. Complete clinical resolution was reported in all patients with available follow-up, and no XO-related mortality occurred. XO demonstrates non-specific clinical and radiographic features that often mimic bone malignancy. Surgical management appears to be both diagnostically and therapeutically effective in most reported cases, although standardized antimicrobial treatment strategies remain undefined.

## Introduction and background

Xanthogranulomatous osteomyelitis (XO) is a rare, non-neoplastic, chronic inflammatory bone disorder characterized by the presence of lipid-laden (foamy) macrophages, multinucleated giant cells, lymphocytes, and plasma cells within the affected tissue [1]. Though most commonly associated with organs, such as the kidney and gallbladder, skeletal involvement is exceedingly rare [1,2]. Due to its rarity, XO is often mistaken for bone tumors, other inflammatory bone conditions, or histiocytic disorders such as Langerhans cell histiocytosis or Erdheim-Chester disease [1]. Its radiologic appearance is frequently destructive, with features resembling neoplastic lesions or chronic osteomyelitis, potentially contributing to diagnostic delays or inappropriate management [3,4].

Histopathologic confirmation demonstrating foamy macrophages (histiocytes), multinucleated giant cells, and plasma cells is necessary to distinguish XO from other aggressive bone lesions [1,3,4]. Cultures may identify causative organisms, most commonly *Staphylococcus aureus*; however, many cases remain culture-negative, and culture yield may be reduced by prior antibiotic exposure [1]. Treatment strategies for XO vary due to the absence of standardized guidelines [1,2]. Management typically involves a combination of surgical debridement and empiric or targeted antimicrobial therapy; however, some cases have been resolved with surgery or antibiotics alone [5].

This systematic review synthesizes all histologically confirmed XO cases in the literature to clarify their clinical features, diagnostic approaches, therapeutic management, and clinical outcomes. Our findings may help clinicians identify and manage this rare condition more effectively.

## Review

Methods

Study Design and Objective

This study was conducted as a systematic review in accordance with the Preferred Reporting Items for Systematic Reviews and Meta-Analyses (PRISMA) guidelines. The objective was to synthesize all English-language case reports and case series published between January 1984 and May 2025 that described the diagnosis, clinical manifestations, and treatment of xanthogranulomatous osteomyelitis. Given the rarity of this disease, only case-based literature was eligible for inclusion. The final PubMed search was completed in May 2025, and no additional databases (e.g., Embase or Scopus) were searched. Titles and abstracts were independently screened by two reviewers, followed by a full-text review for eligibility; discrepancies were resolved by consensus.

Search Strategy and Data Sources

We conducted a comprehensive search of published case reports and series in PubMed without time restrictions, but included only English-language full-text reports. The search strategy combined title/abstract keywords using Boolean operators to maximize sensitivity and specificity. The terms “Xanthogranulomatous osteomyelitis,” “Xanthogranulomatous,” and “osteomyelitis” were combined using Boolean operators (AND, OR) to capture all relevant case reports and case series. The specific search strategy is provided below (Table 1).

**Table 1:** Search terms and Boolean operators used to identify cases of xanthogranulomatous osteomyelitis from the literature.

Serial no.	Search terms
#1	("Xanthogranulomatous osteomyelitis"[Title/Abstract])
#2	("Xanthogranulomatous"[Title/Abstract])
#3	("osteomyelitis"[Title/Abstract])
#4	#1 OR #2 AND #3
Final combined search	("Xanthogranulomatous osteomyelitis" [Title/Abstract] OR ("Xanthogranulomatous" [Title/Abstract] AND "osteomyelitis" [Title/Abstract]))

Study Characteristics

All included studies were case reports or small case series published between 1984 and 2025 [1-20]. All articles were published in English and provided sufficient clinical, diagnostic, and therapeutic detail.

Inclusion and Exclusion Criteria

English-language full-text case reports and case series on human subjects were eligible for inclusion. Articles had to describe the clinical presentation, diagnosis, or treatment of xanthogranulomatous osteomyelitis to be included in this study. Non-human studies, letters to the editor, and studies unrelated to the diagnosis or management of xanthogranulomatous osteomyelitis were excluded. Studies that failed to meet quality or relevance thresholds were also excluded to ensure methodological rigor.

Outcome Measures

The primary outcomes assessed in this systematic review were symptom resolution, surgical versus conservative intervention, and mortality. Outcomes were extracted from each included study based on the reported clinical follow-up, imaging assessments, and intraoperative findings.

Data Extraction and Analysis

Study selection followed a two-step screening process. Initially, titles and abstracts were reviewed to assess relevance. Articles that met the inclusion criteria underwent full-text review to confirm eligibility. The selection process is outlined in the PRISMA flow diagram. Data extraction was performed systematically using a standardized collection form to ensure consistency and completeness. The extracted information included the following: (1) key bibliographic details (author(s) and year of publication); (2) patient-specific data (age, sex, immune status); (3) diagnostic findings (bone involvement, clinical presentation, diagnostic methodology {culture, histopathology, imaging findings}); (4) treatment data (antimicrobial therapy type and duration, and surgical versus conservative management); (5) outcomes (mortality, recurrence, resolution).

Data Synthesis and Analysis

Due to the rarity of xanthogranulomatous osteomyelitis, a meta-analysis was not feasible. A structured narrative synthesis was therefore performed, with findings grouped into clinical presentation, diagnostic strategies, therapeutic approaches, and clinical outcomes.

Assessment of Bias

We assessed the risk of bias using the Joanna Briggs Institute (JBI) Critical Appraisal Checklist. This validated tool evaluates potential sources of bias and methodological limitations to determine the reliability of individual case studies. Each article was scored on an eight-point scale as follows: scores of 7-8 were classified as high quality, 4-6 as moderate quality, and ≤3 as low quality. To ensure the integrity of this review, only studies rated as moderate or high quality were included [6].

Results

Study Selection

**Figure 1:**
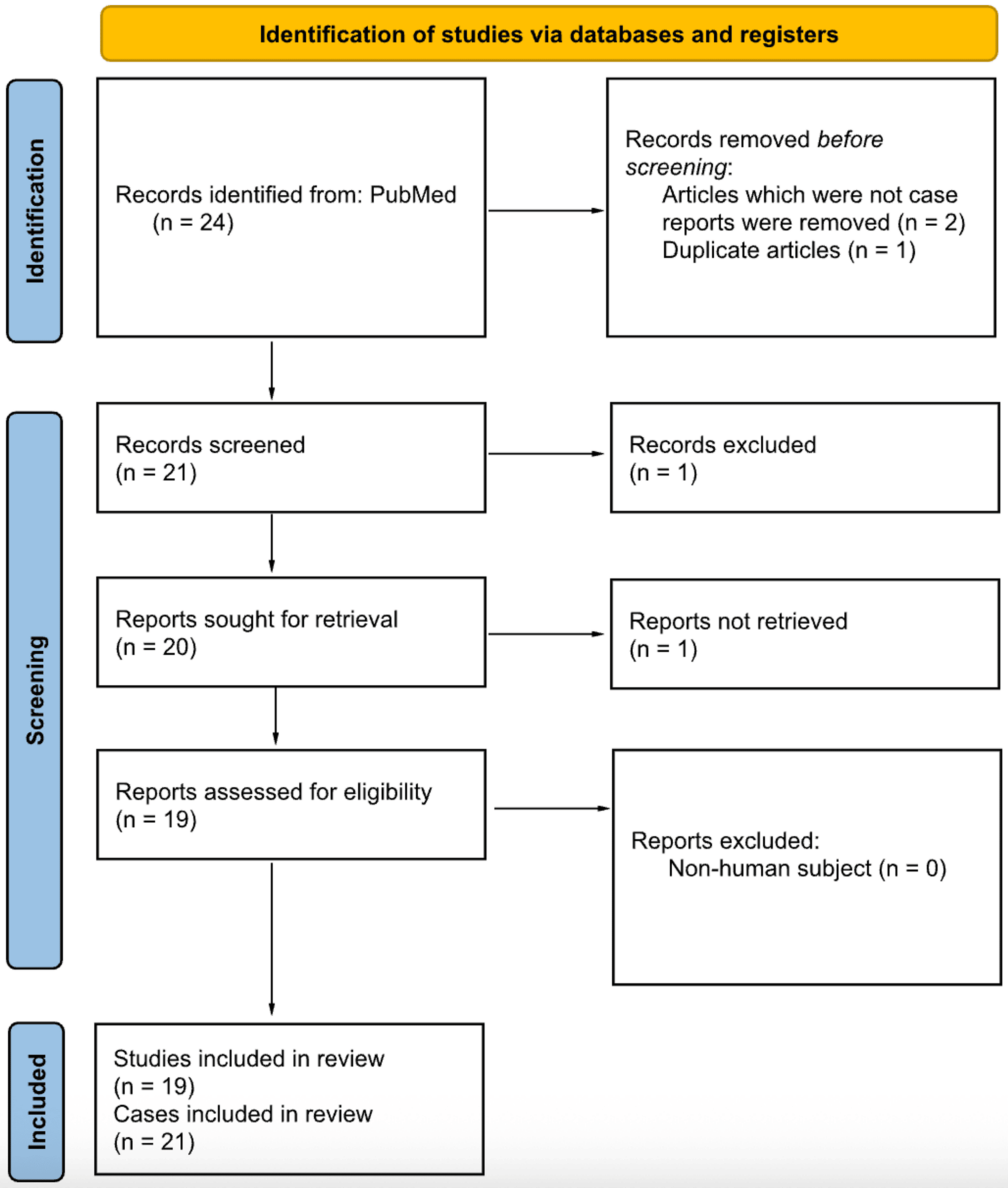
PRISMA flow diagram. Flow of records through the systematic review process - records identified, screened, assessed for eligibility, and included in the final dataset. PRISMA: Preferred Reporting Items for Systematic Reviews and Meta-Analyses

A total of 24 records were identified through database searching. After applying the inclusion and exclusion criteria, 19 studies were included in the final analysis, comprising 21 patients [1-5,7-20]. The included studies were published between 1984 and 2025. The study selection process is summarized in Figure 1. The JBI critical appraisal results are summarized in Table 2. The summary of extracted data from the included studies is presented in Table 3.

**Table 2:** JBI critical appraisal summary. Assessment of study quality using the Joanna Briggs Institute Critical Appraisal Checklist for Case Reports and Case Series. Corresponding references for all included studies are listed in the references section. Q1: Were the patient's demographic characteristics clearly described? Q2: Was the patient's history clearly described and presented as a timeline? Q3: Was the current clinical condition of the patient on presentation clearly described? Q4: Were the diagnostic tests or assessment methods and the results clearly described? Q5: Were the intervention(s) or treatment procedure(s) clearly described? Q6: Was the post-intervention clinical condition clearly described? Q7: Were adverse events (harms) or unanticipated events identified and described? Q8: Does the case report provide takeaway lessons? Y: yes; N: no; JBI: Joanna Briggs Institute

JBI critical appraisal checklist for case reports								
Studies	Q1	Q2	Q3	Q4	Q5	Q6	Q7	Q8
Cozzutto (1984) - Case 1 [16]	Y	Y	Y	Y	Y	Y	Y	N
Cozzutto (1984) - Case 2 [16]	Y	Y	Y	Y	Y	Y	Y	N
Vankalakunti et al. (2007) [17]	Y	Y	Y	Y	Y	Y	Y	N
Kamat et al. (2011) [18]	Y	Y	Y	Y	Y	Y	Y	N
Borjian et al. (2012) [14]	Y	Y	Y	Y	Y	Y	Y	N
Wang et al. (2014) - Case 1 [10]	Y	Y	Y	Y	N	N	N	N
Wang et al. (2014) - Case 2 [10]	Y	Y	Y	Y	N	N	N	N
Singh et al. (2015) [3]	Y	Y	Y	Y	Y	Y	Y	N
Kaneuchi et al. (2015) [9]	Y	Y	Y	Y	Y	Y	Y	N
Baisakh et al. (2016) [7]	Y	Y	Y	Y	N	N	N	Y
Cheema et al. (2017) [5]	Y	Y	Y	Y	Y	Y	Y	N
Solooki et al. (2019) [1]	Y	Y	Y	Y	Y	Y	Y	N
Pathak et al. (2019) [12]	Y	Y	Y	Y	Y	Y	Y	N
Scicluna et al. (2020) [11]	Y	Y	Y	Y	Y	Y	Y	N
Perera et al. (2022) [2]	Y	Y	Y	Y	Y	Y	Y	N
Yadav et al. (2023) [4]	Y	Y	Y	Y	Y	Y	Y	N
Bae et al. (2023) [20]	Y	Y	Y	Y	Y	Y	Y	N
Choi et al. (2024) [8]	Y	Y	Y	Y	Y	Y	Y	N
Lee and Moon (2024) [15]	Y	Y	Y	Y	Y	Y	Y	N
Mohanan et al. (2025) [13]	Y	Y	Y	Y	Y	Y	Y	N
Nair et al. (2025) [19]	Y	Y	Y	Y	Y	Y	Y	N

**Table 3:** Data extraction summary. Condensed summary of reported cases of xanthogranulomatous osteomyelitis, including patient demographics, immune status, anatomical involvement, diagnostic confirmation, management strategies, and outcomes. Corresponding references for all included studies are listed in the references section. IC: immunocompetent; IM: immunocompromised; S + Abx: surgical intervention with antibiotic therapy; S ± Abx: surgical intervention, but antibiotic therapy not specified post-operatively; S + AF: surgical intervention with antifungal therapy; Abx: antibiotic therapy alone

Studies	Age/sex	Immune status	Bone(s)	Diagnostic confirmation	Management	Outcome
Cozzutto (1984) Case 1	5/M	IC	1st rib	Histopathology	S + Abx	Resolution (no recurrence)
Cozzutto (1984) Case 2	14/M	IC	Tibia	Histopathology	S + Abx	Resolution (no recurrence)
Vankalakunti et al. (2007)	50/F	IC	Right ulna	Histopathology + imaging	S	No mortality; lost to follow-up
Kamat et al. (2011)	13/M	IC	Right tibia	Culture + histopathology + imaging	S ± Abx	Resolution (no recurrence)
Borjian et al. (2012)	14/M	Not reported	Right humerus, left fibula	Culture + histopathology + imaging	Abx	Resolution (no recurrence)
Wang et al. (2014) Case 1	45/M	Not reported	Left 3rd rib	Histopathology + imaging	S	Resolution (no recurrence)
Wang et al. (2014) Case 2	46/M	Not reported	Right 8th rib, left 5th rib	Histopathology + imaging	Not reported	Not reported
Singh et al. (2015)	65/F	IC	Femur	Histopathology + imaging	S + Abx	Resolution (no recurrence)
Kaneuchi et al. (2015)	36/F	IC	Tibia	Histopathology + imaging	S	Resolution (no recurrence)
Baisakh et al. (2016)	21/F	IC	Tibia, femur	Histopathology + imaging	Not reported	Not reported
Cheema et al. (2017)	5/F	IM	Humerus	Culture + histopathology + imaging	S + Abx	Resolution (no recurrence)
Solooki et al. (2019)	15/M	IC	Tibia	Culture + histopathology + imaging	Abx	Resolution (no recurrence)
Pathak et al. (2019)	50/F	IC	Left femur	Culture + histopathology + imaging	S + Abx	Resolution (no recurrence)
Scicluna et al. (2020)	20/M	IM	Right pubic bone	Histopathology + imaging	Immunosuppressive therapy (no surgery)	Resolution (no recurrence)
Perera et al. (2022)	65/F	IC	C5-C7 vertebrae	Histopathology + imaging	S	Resolution (no recurrence)
Yadav et al. (2023)	64/M	IC	Temporal bone	Histopathology + imaging	S + Abx	Resolution (no recurrence)
Bae et al. (2023)	81/F	IC	Right mandibular body	Histopathology + imaging	S + Abx	Resolution (no recurrence)
Choi et al. (2024)	41/M	IM	Sternoclavicular joint (clavicle, sternum)	Histopathology + imaging	S	Resolution (no recurrence)
Lee and Moon (2024)	23/F	IC	Right pubic bone	Culture + histopathology + imaging	S + AF	Resolution (no recurrence)
Mohanan et al. (2025)	42/F	IC	Left tibia	Histopathology + imaging	S	Resolution (no recurrence)
Nair et al. (2025)	62/M	IC	Right radius	Histopathology + imaging	S	Resolution (no recurrence)

Patient Demographics

Among the 21 patients, 11 (52.4%) were male, and 10 (47.6%) were female. Patient ages ranged from five to 81 years. Immune status was reported in 18 cases (85.7%), of which 15 patients (71.4%) were immunocompetent, and three patients (14.3%) were immunocompromised.

Anatomic Distribution

Xanthogranulomatous osteomyelitis was reported across 25 skeletal sites in 21 patients. The tibia was the most frequently involved bone (24%), followed by the ribs (16%) and the femur (12%). Additional sites included the humerus (8%), pelvis (8%), and single cases (4%) involving the spine, radius, ulna, temporal bone, mandible, clavicle, sternum, and fibula. Appendicular skeletal involvement accounted for 17 of 25 sites (68%), while axial skeletal involvement accounted for eight of 25 sites (32%).

Clinical Presentation

XO has been reported in both immunocompetent and immunocompromised individuals, although several cases have occurred in patients with underlying immunosuppression or chronic infection. Bone pain was the most frequently reported presenting symptom, occurring in 17 of 21 patients (81%). Swelling or mass-like lesions were reported in 11 cases (52.4%). Systemic symptoms, including fever or chills, were present in seven patients (33.3%), whereas localized inflammatory findings, such as erythema or warmth, were noted in four cases (19%). Tenderness on physical examination was described in six patients (28.6%). Functional impairment was less common, with the following findings: gait disturbance or mobility limitation was reported in three cases (14.3%), and restricted range of motion in three cases (14.3%).

Diagnostic Strategies

Histopathologic evaluation was performed in 100% of cases and consistently demonstrated lipid-laden (foamy) macrophages with granulomatous inflammation, confirming the diagnosis of xanthogranulomatous osteomyelitis. Imaging studies were commonly used, including MRI in 13 cases (61.9%), plain radiography in 12 cases (57.1%), and CT in eight cases (38.1%), all demonstrating lytic, destructive, or expansile osseous lesions. Microbiologic cultures were obtained in six cases (28.6%), with *Staphylococcus aureus* identified in 66.7% of cases. The sampling technique was explicitly described as a biopsy in one case, while the remaining cases reported histopathologic findings without specifying the sampling technique.

Treatment Approaches

Surgical management was performed in 16 of 21 patients (76.2%). Conservative, non-operative management was reported in three cases (14.3%), while the treatment strategy was not clearly specified in two cases (9.5%). Antimicrobial therapy was administered to eight patients (38.1%), with antibiotic selection and duration reported variably. Cephalosporins were the most frequently used agents (six cases), followed by lincosamides (two cases) and penicillins (two cases). Fluoroquinolones and oxazolidinones were each used in one case. Antifungal therapy was reported in one case, corresponding to a culture-confirmed Aspergillus infection.

Clinical Outcomes

Complete clinical resolution was reported in 100% of patients with available follow-up data. No deaths were attributed to xanthogranulomatous osteomyelitis. Recurrence data were reported inconsistently, and the duration of follow-up varied across cases.

Discussion

This systematic review summarizes all published English-language case reports and case series of xanthogranulomatous osteomyelitis (XO) from 1984 to 2025. Although rare, similarities in clinical, radiographic, and histopathological features between XO and primary bone malignancies, as illustrated by the present cases, underscore the diagnostic challenge posed by XO. Most commonly, XO is diagnosed as a destructive mass-forming bone lesion associated with non-specific symptoms, which often results in concern for tumors or severe infection. This review synthesizes the characteristics of XO and identifies trends in diagnostic evaluation, treatment options, and prognosis.

The distribution of skeletal site involvement appeared extensive, with a predilection for long bones (femur, tibia); however, other skeletal sites (vertebrae, ribs, pelvis, craniofacial area) were also affected. The wide anatomic distribution further complicates diagnosis, as many lesions occur at sites that are typically associated with malignant disease. XO typically appears as an aggressive osteolytic lesion with cortical destruction, marrow replacement, and an enhancing extraosseous soft-tissue component on CT and MRI. These findings closely resemble primary bone malignancy or metastatic disease and do not demonstrate imaging features that reliably distinguish infection. Additionally, in one case, FDG uptake on PET further increased suspicion for neoplasia [10]. As a result, imaging findings alone are insufficient for definitive diagnosis, and histopathologic evaluation remains necessary for definitive differentiation.

Histopathological analysis played a significant role in establishing a definitive diagnosis of XO by revealing lipid-laden macrophages, chronic inflammatory infiltrates, and necrotic bone tissue [12]. These markers allowed for differentiation of XO from other neoplastic and inflammatory diseases [1]. Evidence from microbiological cultures was inconsistent; however, among the limited number of cases with positive cultures, *Staphylococcus aureus* was the most frequently isolated organism. However, the low overall culture yield and frequent culture-negative cases preclude firm conclusions regarding microbial etiology and suggest that XO is not uniformly infection-driven [2]. The presence of immunosuppression in a subset of reported cases suggests that dysregulated macrophage-mediated inflammation may contribute to XO pathogenesis rather than a purely infectious process [5,8,11].

Surgical management was performed in the majority of cases and varied according to anatomic location, structural integrity, and the presence of active infection [1-5,8-10,12,13,15-20]. For contained long-bone lesions, intralesional curettage with or without bone grafting was most frequently used, whereas internal fixation or segmental excision was performed in cases with structural instability, including pathological fracture or extensive cortical destruction [3,9,18]. Axial and anatomically complex disease required site-specific procedures, including vertebrectomy with anterior column reconstruction for spinal involvement and modified radical mastoidectomy for temporal bone lesions [2,4]. Lesions that mimicked malignancy, particularly in the rib, sternoclavicular region, and mandible, were managed with en bloc or wide excision [8,10,16,20]. Finally, a small subset of patients were treated non-operatively [1,14]. Overall, the extent of surgery was determined by biomechanical and anatomic factors rather than the histopathologic diagnosis alone [1,14].

The results of this systematic review indicate that XO is a non-neoplastic inflammatory process that frequently mimics malignancy on clinical and radiologic evaluation [4,9,14]. Histopathologic confirmation, therefore, remains essential for establishing the diagnosis and guiding management, potentially preventing unnecessary oncologic interventions [1,12]. Histopathologic differential diagnoses include chronic osteomyelitis, Langerhans cell histiocytosis, Erdheim-Chester disease, and other granulomatous or histiocytic inflammatory processes that may demonstrate similar histologic features. Although antimicrobial therapy was used variably, the heterogeneity of regimens and the limited microbiologic confirmation preclude evidence-based recommendations regarding its independent therapeutic role. Future accumulation of culture-positive cases with standardized reporting may clarify whether targeted antimicrobial therapy can serve as an effective adjunct to surgical management in selected patients. Increased awareness and consistent reporting of diagnostic and therapeutic strategies may improve recognition of this rare entity and help refine evidence-based treatment algorithms.

Limitations

This review is limited by the rarity of xanthogranulomatous osteomyelitis and the nature of existing literature. All included studies were single-case reports or case series, precluding robust statistical analysis or generalizability. Reporting quality was inconsistent, with some studies omitting key clinical, microbiological, or follow-up data. Finally, this review was limited to a single database search, which may have resulted in missed cases. Despite these limitations, this is the most comprehensive synthesis of XO to date and highlights recurring diagnostic and therapeutic themes.

## Conclusions

Xanthogranulomatous osteomyelitis is a rare, underrecognized condition that can mimic malignancy on imaging and clinical examination. Histopathological confirmation is essential for diagnosis. Surgical intervention remains the mainstay of treatment, with generally favorable outcomes. Reported antimicrobial use was heterogeneous and inconsistently described, precluding conclusions regarding optimal regimens. Increased awareness and consistent reporting practices are needed to inform future management and clarify the risk of recurrence.
